# Crystal structure of *fac*-{5-[(hexyl­aza­nium­yl)meth­yl]-2-(pyridin-2-yl)phenyl-κ^2^ *N*,*C* ^1^}bis­[2-(pyridin-2-yl)phenyl-κ^2^ *N*,*C* ^1^]iridium(III) chloride

**DOI:** 10.1107/S2056989018012811

**Published:** 2018-09-14

**Authors:** Sureemas Meksawangwong, Suwadee Jiajaroen, Kittipong Chainok, Waraporn Pinyo, Filip Kielar

**Affiliations:** a Department of Chemistry, Faculty of Science, Naresuan University, Muang, Phitsanulok 65000, Thailand; b Division of Chemistry, Faculty of Science and Technology, Thammasat University, Klong Luang, Pathum Thani 12121, Thailand; c Materials and Textile Technology, Faculty of Science and Technology, Thammasat University, Klong Luang, Pathum Thani 12121, Thailand; d NSTDA Characterization and Testing Center, Thailand Science Park, Klong Luang, Pathum Thani 12120, Thailand

**Keywords:** crystal structure, hydrogen bonds, iridium(III)

## Abstract

The asymmetric unit of the title compound, *fac*-[Ir(C_11_H_8_N)_2_(C_18_H_24_N_2_)]Cl or *fac*-[Ir(ppy)_2_(Hppy-NC_6_)]Cl, contains two [Ir(ppy)_2_(ppy-NC_6_)](H^+^) cations, two Cl^−^ anions and disordered solvent. In each complex mol­ecule, the Ir^III^ ion is coordinated by two *C*,*N*-bidentate 2-(pyridin-2-yl)phenyl ligands and one *C*,*N*-bidentate *N*-[4-(pyridin-2-yl)benz­yl]hexan-1-aminium ligand, leading to a distorted *fac*-octa­hedral coordination environment. In the crystal, the mol­ecules are linked by N—H⋯Cl, C—H⋯π and π–π inter­actions, forming a three-dimensional supra­molecular structure. The hexyl group of one mol­ecule is disordered over two orientations with a refined occupancy ratio of 0.412 (13):0.588 (13). The acetone and hexane solvent mol­ecules were found to be highly disordered and their contribution to the scattering was masked using the solvent-masking routine smtbx.mask in *OLEX2* [Rees *et al.* (2005 ▸). *Acta Cryst*. D**61**, 1299–1301]. These solvent mol­ecules are not considered in the given chemical formula and other crystal data.

## Abstract 2

The asymmetric unit of the title compound, *fac*-[Ir(C_11_H_8_N)_2_(C_18_H_24_N_2_)]Cl or *fac*-[Ir(ppy)_2_(Hppy-NC_6_)]Cl, contains two [Ir(ppy)_2_(ppy-NC_6_)](H^+^) cations, two Cl^−^ anions and a disordered solvent. In each complex mol­ecule, the Ir^III^ ion is coordinated by two *C*,*N*-bidentate 2-(pyridin-2-yl)phenyl ligands and one *C*,*N*-bidentate *N*-[4-(pyridin-2-yl)benz­yl]hexan-1-aminium ligand, leading to a distorted *fac*-octa­hedral coordination environment. In the crystal, the mol­ecules are linked by N—H⋯Cl, C—H⋯π and π–π inter­actions, forming a three-dimensional supra­molecular structure.

## Chemical context   

Luminescent iridium complexes have attracted a significant amount of inter­est over the past decades as they have been shown to possess potential for use in a number of applications such as in organic-light emitting devices (OLED), cellular imaging and photoredox catalysis (You, 2013 ▸; You & Nam, 2012 ▸; König, 2017 ▸; Caporale & Massi, 2018 ▸). The beneficial photophysical properties of these complexes, which are at the core of their potential utilization, arise both from the properties of the Ir^3+^ ion and its coordination environment. The large spin–orbit coupling constant of iridium ensures efficient involvement of triplet excited states in the photophysical properties, which results in luminescent lifetimes in the microsecond regime (Ladouceur, S. & Zysman-Colman, 2013 ▸; Zanoni *et al.*, 2015 ▸; Thorp-Greenwood *et al.*, 2012 ▸). This is significantly longer than for fluorescence from organic fluoro­phores, a benefit for imaging applications, yet also much shorter than phospho­rescence lifetimes of organic phosphors, which is important for OLED applications. The NC cyclo­metalating ligands such as 2-phenyl­pyridine usually used to chelate the iridium center provide strong ligand fields, which result in lifting of the unfilled metal-based orbitals above the π* orbitals of the ligands, thus eliminating metal-centered transitions from the photophysical properties (You & Nam, 2012 ▸). Thus, the usual electronic transitions present in the photochemistry of luminescent iridium complexes have charge–transfer characteristics such as metal-to-ligand charge transfer (MLCT) or ligand-to-ligand charger transfer (LLCT).
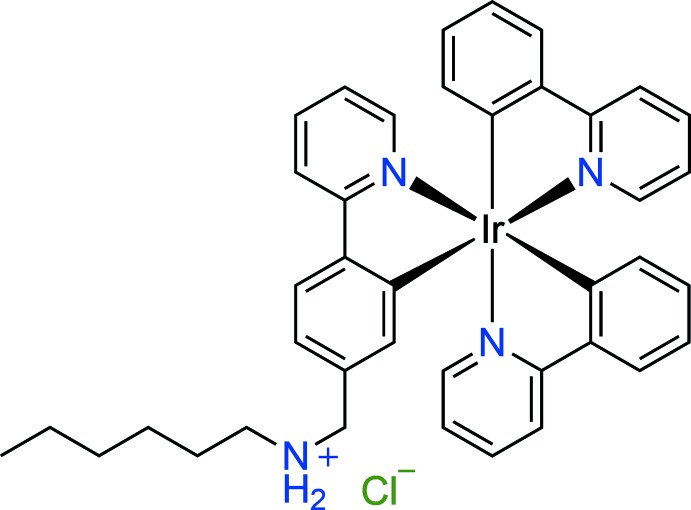


Luminescent iridium complexes can be divided into several distinct classes, one of which is tris-cyclo­metalated complexes. These complexes contain three cyclo­metalating NC ligands such 2-phenyl­pyridine (ppy) and the prototypical example of this structural class is [Ir(ppy)_3_] (You & Nam, 2012 ▸). These complexes usually exhibit good photophysical properties. However, their use in cellular imaging is limited as they do not seem to be very readily taken up by cells (Fernández-Moreira *et al.*, 2010 ▸; Steunenberg *et al.*, 2012 ▸; Ho *et al.*, 2012 ▸). It has been noted that this problem can be alleviated by introducing protonatable groups into their structures, which helps them to become positively charged and thus be better taken up by cells (Kando *et al.*, 2015 ▸). We have recently reported two simple derivatives of the prototypical structure mentioned above, which contain an amino­alkyl side chain on one of the ppy ligands (Sansee *et al.*, 2016 ▸). The complexes differ only in the length of the alkyl chain, one being butyl while the other one is dodecyl. Both complexes are capable of staining live cells in fluorescence microscopy experiments. Furthermore, the complexes also exhibit ratiometric response to pH, which depends on their structure and is attributed to changes in their aggregation status. Several further analogues of these complexes are currently being investigated in order to obtain more detailed knowledge of the relationship between the structure of these compounds and their photophysical properties. The complex reported herein is one of these further compounds studied for this purpose.

## Structural commentary   

**Figure 1:**
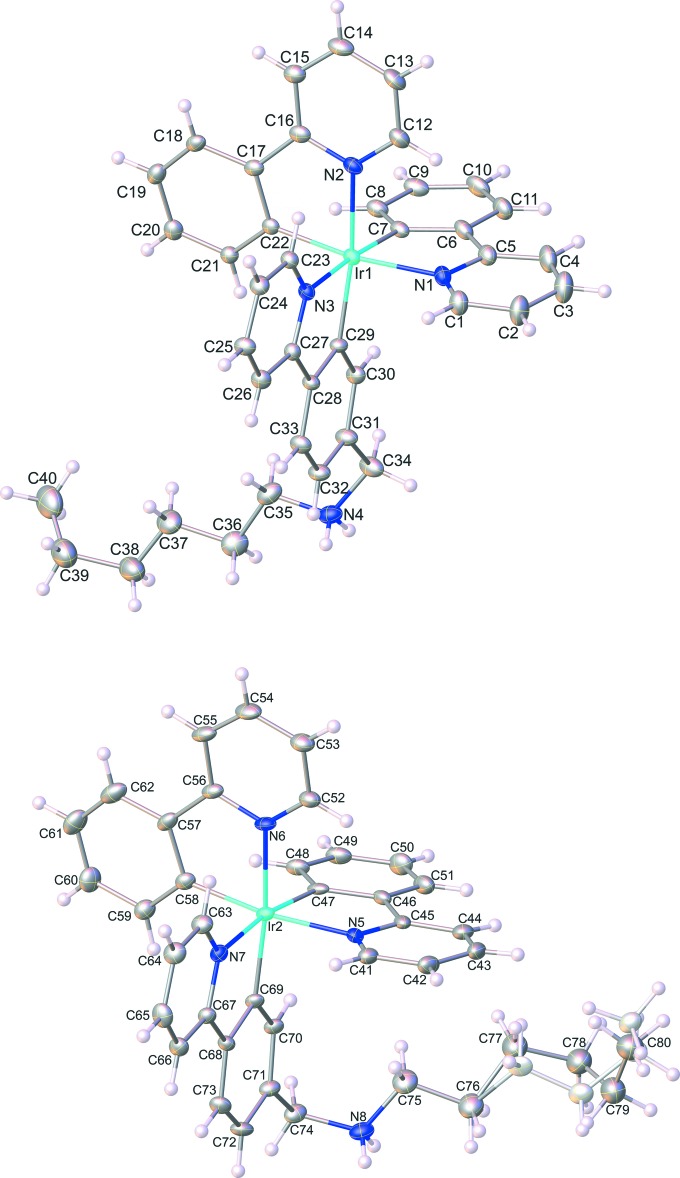
A view of the mol­ecular structures of the two independent cationic mol­ecules of the title compound, with the atom labelling. Displacement ellipsoids are drawn at the 35% probability level.

The asymmetric unit of the title compound contains two [Ir(ppy)_2_(Hppy-NC_6_)]^+^ cations, two Cl^−^ anions and disordered solvent mol­ecules. In each complex mol­ecule, the Ir^III^ ion is coordinated by two *C*,*N*-bidentate ppy ligands and one *C*,*N*-bidentate Hppy-NC_6_ ligand, leading to a distorted *fac*-octa­hedral coordination environment as shown in Fig. 1 ▸. The Ir—C and Ir—N bond lengths in the title compound range from 2.010 (6) to 2.036 (5) Å and 2.105 (5) to 2.144 (4) Å, respectively, whereas the bond angles in the [IrN_3_C_3_] octa­hedral core vary from 79.1 (2) to 172.1 (2)°. These structural features are typical of related iridium(III) complexes containing *C*,*N*-donor set ligands (Steunenberg *et al.*, 2012 ▸). The current mol­ecule is isostructural with the butyl equivalent and displays similar packing and voids (see *Refinement* section) in the solid state. Full details of this structure have been published by Sansee *et al.* (2016 ▸).

## Supra­molecular features   

**Figure 2:**
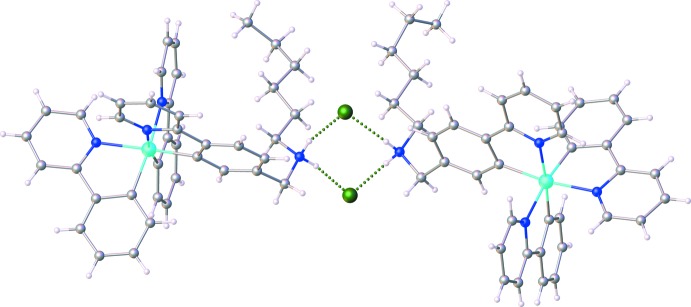
A perspective view of the title compound, showing the inter­molecular N—H⋯Cl hydrogen bonds (dotted lines) between the two independent mol­ecules.

**Figure 3:**
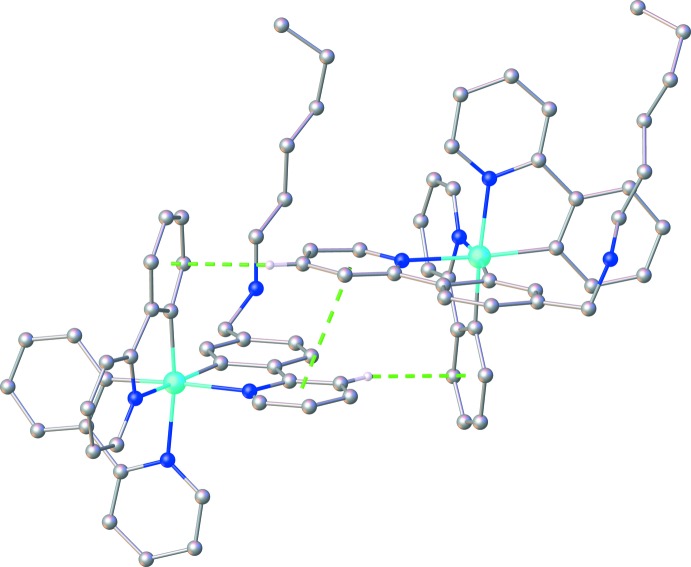
A perspective view showing the parallel fourfold phenyl embrace in the title compound.

In the crystal, pairs of cationic [Ir(ppy)_2_(Hppy-NC_6_)]^+^ complex mol­ecules are linked through N—H⋯Cl hydrogen bonds (Table 1 ▸) between the amino groups of the ppy-NC_6_ ligands and chloride anions (Fig. 2 ▸) with an Ir⋯Ir separation of 15.8207 (7) Å. Simultaneously, pairs of cationic complexes with an Ir⋯Ir separation of 8.5468 (4) Å (Fig. 3 ▸) also inter­act with each other *via* a parallel fourfold phenyl embrace (Dance & Scudder, 1996 ▸), which contains one π–π stacking [centroid-to-centroid distance between the N3/C23–C27 and N7/C63–C67 rings = 3.682 (3) Å; dihedral angle = 6.5 (5)°] and two edge-to-face (phen­yl)-C—H⋯π(phen­yl) inter­actions (H26⋯*Cg*5 = 2.79 Å and H65⋯*Cg*3 = 2.85 Å; *Cg*5 and *Cg*3 are the centroids of the C57–C62 and C17–C22 rings, respectively). Numerous weak (phen­yl)-C—H⋯π(phen­yl) and (methyl­ene)-C—H⋯π(phen­yl) are observed with H⋯centroid distances ranging from 2.79 to 3.12 Å (Table 1 ▸). In addition, a comparison of the effect of the alkyl chain length between the ppy-NC6 in the title compound and the related complex with ppy-NC4 (Sansee *et al.*, 2016 ▸) on the packing arrangement suggests that the key inter­molecular inter­actions (N—H⋯Cl, C—H⋯π and π–π) remain the same.

## Photophysical properties   

**Figure 4:**
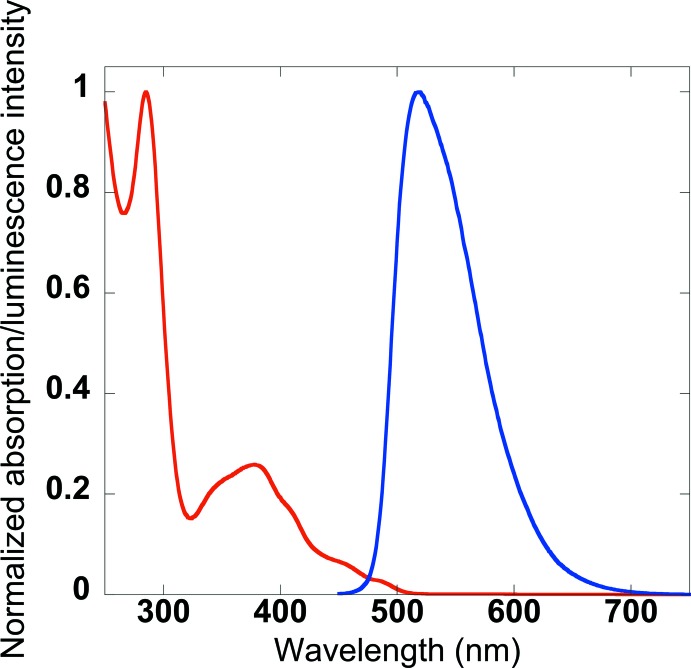
The photophysical properties of the title compound.

The photophysical properties of the title compound have also been investigated in di­chloro­methane solution and the results can be seen in Fig. 4 ▸, which shows normalized absorption and emission spectra. The spectra exhibit the expected features, which are analogous to those of the parent complex and the complexes previously reported by our group. The absorption spectra can be roughly divided into three portions. The first portion lies between 250 and 320 nm and is mainly attributed to ligand-based π to π* transitions. The second portion of this spectrum lies between 320 and 430 nm and is attributed to spin-allowed singlet metal-to-ligand charge transfer (^1^MLCT) transition. Finally, the tail of the spectrum extending from 430 nm beyond 500 nm is attributed to spin-forbidden triplet metal-to-ligand charge transfer (^3^MLCT). The emission spectrum exhibits a single unstructured peak centered at 520 nm. The photoluminescence quantum yield has been determined to be 39%.

## Synthesis and crystallization   

All chemicals and reagents were of commercial grade and were used without further purification. The complex *fac*-[Ir(ppy)_2_(Fppy)] [ppy is 2-phenylpyridine and Fppy is 2-(2,4-difluorophenyl)pyridine] was prepared according to a literature proc­edure (Beeby *et al.*, 2003 ▸). ^1^H NMR spectra were recorded on a Bruker Advance 400MHz instrument operating at 400 MHz. The ^13^C NMR spectrum was recorded on the same instrument operating at 100 MHz for carbon. Mass spectra were acquired with an Agilent technologies UHD Accurate-Mass Q-TOF LC–MS instrument model 6540. UV-Visible absorption spectra were recorded using an Analytik Jena 210plus diode array spectrophotometer. Steady-state emission spectra were recorded using Fluoro­max-4 and Fluoro­log spectro­fluoro­meters from Yvon Horiba. Phos­phor­escence lifetime measurements were performed on a DeltaFlexTM instrument equipped with a UV LED (λ_ex_ = 372 nm).

*fac*-[Ir(ppy)_3_(Fppy)] (200 mg, 0.29 mmol), *n*-hexyl­amine (90 µL, 0.44 mmol) and tri­ethyl­amine (40 µL, 0.43 mmol) were suspended in a CH_3_OH/CH_2_Cl_2_ (1:1) mixture (20 mL). The reaction mixture was heated to reflux for 10 h. The solution was left to cool to room temperature and NaBH_4_ (37 mg, 0.58 mmol) was added. The reaction mixture was stirred at room temperature for 20 h. The solvent was removed under vacuum. The residue was dissolved in di­chloro­methane, dried over anhydrous sodium sulfate and filtered. The residue was purified by column chromatography on silica using gradient of methanol (up to 5%) in di­chloro­methane as the eluent. The pure product was isolated as an orange solid (yield 89.5%, 204 mg). Single crystals of the complex suitable for the single crystal X-ray diffraction analysis were grown by slow diffusion of hexane into its solution in acetone.

^1^H NMR (400 MHz, DMSO-*d*6, δ) 8.18 (*d*, *J* = 8.2 Hz, 1H), 8.13 (*d*, *J* = 8.0 Hz, 2H), 7.85–7.70 (*m*, 6H), 7.40–7.50 (*m*, 3H), 7.15–7.06 (*m*, 3H), 7.05 (*d*, *J* = 7.8 Hz, 1H), 6.75–6.85 (*m*, 2H), 6.70–6.65 (*m*, 5H), 3.67 (*s*, 2H), 2.52–2.65 (*m*, 2H), 1.47 (*m*, 2H), 1.32–1.21 (*m*, 6H), 0.85 (*t*, *J* = 6.7 Hz, 3H). ^13^C NMR (100 MHz, DMSO-*d*6, δ) 165.5, 165.0, 161.2, 160.1, 146.8, 144.5, 143.7, 137.5, 137.0, 136.2, 133.0, 129.0, 124.2, 123.1, 122.8, 120.6, 119.7, 119.4, 119.1, 130.2, 130.0, 125.0, 124.1, 122.6, 122.4, 122.2, 122.1, 121.7, 120.4, 120.1, 119.3, 119.0, 118.8, 50.6, 46.1, 30.7, 25.8, 25.5, 21.9, 13.8. HRMS (ES^+^) calculated for C_40_H_40_IrN_4_ (769.2882); found 769.2937. In di­chloro­methane at 298 K, λ_ex_ = 390 nm and λ_em_ = 515 nm while the luminescence lifetime is 70 ns and 1.44 µs, respectively, for aerated and degassed solutions.

## Refinement   

Crystal data, data collection and structure refinement details are summarized in Table 2 ▸. The hydrogen atoms attached to carbon atoms were placed in calculated positions and constrained to ride on their parent with *U*_iso_(H) = 1.2*U*_eq_(C) and a C—H distance of 0.93 Å for aromatic and 0.97 Å for methyl­ene hydrogen atoms. The nitro­gen-bound hydrogen atoms were located in a difference-Fourier map but were refined with a distance restraint of N—H = 0.89 Å with *U*_iso_(H) = 1.2*U*_eq_(N). The hexyl group of one complex is disordered over two orientations with a refined occupancy ratio of 0.412 (13):0.588 (13). Anisotropic displacement parameters of all atoms were restrained using enhanced rigid-bond restraints (RIGU command; Thorn *et al.*, 2012 ▸). All attempts to model disordered acetone or hexane as the solvents used for crystallization failed. Therefore, the solvent-masking routine smtbx.mask (Rees *et al.*, 2005 ▸) was used and found four solvent-accessible voids in the unit cell. Two of them are of 490 Å^3^ in volume and contain an estimated 71 electrons; the other two are of 157 Å^3^ in volume and contain an estimated 60 electrons. These electrons are attributable to four mol­ecules of acetone and two mol­ecules of hexane, which means that there are two mol­ecules of acetone and one mol­ecule of hexane per formula unit present in this structure.

## Supplementary Material

Crystal structure: contains datablock(s) I. DOI: 10.1107/S2056989018012811/pj2058sup1.cif

Structure factors: contains datablock(s) I. DOI: 10.1107/S2056989018012811/pj2058Isup2.hkl

Supporting information file. DOI: 10.1107/S2056989018012811/pj2058Isup3.cdx

CCDC reference: 1867002

Additional supporting information:  crystallographic information; 3D view; checkCIF report

## Figures and Tables

**Table 1:** Hydrogen-bond geometry (Å, °) *Cg*1–*Cg*6 are the centroids of the C6–C11, N2/C12–C16, C17–C22, C46–C51, C57–C62 and C68–C73 rings, respectively.

*D*—H⋯*A*	*D*—H	H⋯*A*	*D*⋯*A*	*D*—H⋯*A*
N4—H4*A*⋯Cl2^i^	0.89	2.30	3.172 (6)	168
N4—H4*B*⋯Cl1^i^	0.89	2.26	3.142 (6)	172
N8—H8*A*⋯Cl2	0.89	2.21	3.073 (6)	165
N8—H8*B*⋯Cl1	0.89	2.16	3.044 (6)	171
C20—H20⋯*Cg*1^ii^	0.93	3.12	3.497 (7)	145
C24—H24⋯*Cg*4^iii^	0.93	2.89	3.532 (7)	139
C26—H26⋯*Cg*5	0.93	2.79	3.645 (7)	158
C34—H34*B*⋯*Cg*3^ii^	0.97	2.91	3.422 (7)	160
C37—H37⋯*Cg*1^ii^	0.97	3.01	3.818 (7)	141
C49—H49⋯*Cg*2^iii^	0.93	3.07	3.705 (7)	145
C53—H53⋯*Cg*6^iii^	0.93	3.10	3.692 (7)	135
C65—H65⋯*Cg*3	0.93	2.86	3.530 (7)	135

Symmetry codes: (i) 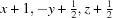; (ii) 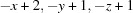; (iii) 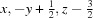.

**Table 2:** Experimental details

Crystal data	
Chemical formula	[Ir(C_11_H_8_N)_2_(C_18_H_24_N_2_)]Cl
*M*_r_	804.41
Crystal system, space group	Monoclinic, *P*2_1_/*c*
Temperature (K)	298
*a*, *b*, *c* (Å)	12.1012 (7), 34.267 (2), 18.4681 (11)
β (°)	94.471 (2)
*V* (Å^3^)	7634.9 (8)
*Z*	8
Radiation type	Cu *K*α
μ (mm^−1^)	7.64
Crystal size (mm)	0.18 × 0.1 × 0.1

Data collection	
Diffractometer	Bruker D8 VENTURE
Absorption correction	Multi-scan (*SADABS*; Bruker, 2016 ▸)
*T*_min_, *T*_max_	0.635, 0.734
No. of measured, independent and observed [*I* > 2σ(*I*)] reflections	110242, 15577, 14944
*R*_int_	0.056
(sin θ/λ)_max_ (Å^−1^)	0.626

Refinement	
*R*[*F* ^2^ > 2σ(*F* ^2^)], *wR*(*F* ^2^), *S*	0.052, 0.135, 1.07
No. of reflections	15577
No. of parameters	869
No. of restraints	142
H-atom treatment	H-atom parameters constrained
Δρ_max_, Δρ_min_ (e Å^−3^)	3.11, −1.29

Computer programs: *APEX3* and *SAINT* (Bruker, 2016 ▸), *SHELXT* (Sheldrick, 2015*a* ▸), *SHELXL2014* (Sheldrick, 2015*b* ▸) and *OLEX2* (Dolomanov *et al.*, 2009 ▸).

## supplementary crystallographic information

### Crystal data

**Table:**

[Ir(C_11_H_8_N)_2_(C_18_H_24_N_2_)]Cl	*F*(000) = 3216
*M**_r_* = 804.41	*D*_x_ = 1.400 Mg m^−^^3^
Monoclinic, *P*2_1_/*c*	Cu *K*α radiation, λ = 1.54178 Å
*a* = 12.1012 (7) Å	Cell parameters from 9555 reflections
*b* = 34.267 (2) Å	θ = 4.4–74.7°
*c* = 18.4681 (11) Å	µ = 7.64 mm^−^^1^
β = 94.471 (2)°	*T* = 298 K
*V* = 7634.9 (8) Å^3^	Block, light yellow
*Z* = 8	0.18 × 0.1 × 0.1 mm

### Data collection

**Table:**

Bruker D8 VENTURE diffractometer	15577 independent reflections
Radiation source: X-ray tube, Micro focus tube	14944 reflections with *I* > 2σ(*I*)
Graphite monochromator	*R*_int_ = 0.056
Detector resolution: 10.4167 pixels mm^-1^	θ_max_ = 74.8°, θ_min_ = 4.5°
φ and ω scans	*h* = −15→15
Absorption correction: multi-scan (SADABS; Bruker, 2016)	*k* = −42→41
*T*_min_ = 0.635, *T*_max_ = 0.734	*l* = −23→23
110242 measured reflections	

### Refinement

**Table:**

Refinement on *F*^2^	Primary atom site location: dual
Least-squares matrix: full	Hydrogen site location: inferred from neighbouring sites
*R*[*F*^2^ > 2σ(*F*^2^)] = 0.052	H-atom parameters constrained
*wR*(*F*^2^) = 0.135	*w* = 1/[σ^2^(*F*_o_^2^) + (0.0495*P*)^2^ + 49.4936*P*] where *P* = (*F*_o_^2^ + 2*F*_c_^2^)/3
*S* = 1.07	(Δ/σ)_max_ = 0.002
15577 reflections	Δρ_max_ = 3.11 e Å^−^^3^
869 parameters	Δρ_min_ = −1.29 e Å^−^^3^
142 restraints	

### Special details

**Table:**

Geometry. All esds (except the esd in the dihedral angle between two l.s. planes) are estimated using the full covariance matrix. The cell esds are taken into account individually in the estimation of esds in distances, angles and torsion angles; correlations between esds in cell parameters are only used when they are defined by crystal symmetry. An approximate (isotropic) treatment of cell esds is used for estimating esds involving l.s. planes.

### Fractional atomic coordinates and isotropic or equivalent isotropic displacement parameters (Å^2^)

**Table:**

	*x*	*y*	*z*	*U*_iso_*/*U*_eq_	Occ. (<1)
Ir1	1.07478 (2)	0.43998 (2)	0.33055 (2)	0.03495 (8)	
Ir2	0.76998 (2)	0.24093 (2)	0.51021 (2)	0.03391 (8)	
Cl1	0.41899 (15)	0.04389 (5)	0.29918 (10)	0.0557 (4)	
Cl2	0.28414 (15)	0.17148 (5)	0.23161 (11)	0.0596 (5)	
N1	1.2374 (4)	0.43256 (16)	0.2959 (3)	0.0437 (12)	
N2	1.0072 (5)	0.45999 (14)	0.2272 (3)	0.0406 (11)	
N3	1.0194 (4)	0.38123 (13)	0.3108 (3)	0.0342 (10)	
N4	1.2417 (5)	0.41718 (18)	0.6886 (3)	0.0549 (15)	
H4A	1.253977	0.391670	0.693468	0.066*	
H4B	1.286575	0.429268	0.721868	0.066*	
N5	0.6118 (4)	0.24667 (16)	0.5517 (3)	0.0377 (10)	
N6	0.8554 (5)	0.26539 (15)	0.6040 (3)	0.0426 (12)	
N7	0.7597 (4)	0.29451 (14)	0.4543 (3)	0.0374 (10)	
N8	0.4565 (5)	0.12975 (17)	0.3354 (3)	0.0529 (14)	
H8A	0.407718	0.145515	0.311668	0.064*	
H8B	0.437654	0.105380	0.323207	0.064*	
C1	1.2805 (6)	0.3999 (2)	0.2736 (4)	0.0588 (18)	
H1	1.238862	0.377185	0.275968	0.071*	
C2	1.3852 (7)	0.3971 (3)	0.2464 (6)	0.078 (3)	
H2	1.412936	0.373439	0.230847	0.094*	
C3	1.4449 (8)	0.4315 (3)	0.2440 (6)	0.084 (3)	
H3	1.515140	0.431231	0.226826	0.101*	
C4	1.4019 (7)	0.4656 (3)	0.2666 (5)	0.071 (2)	
H4	1.441332	0.488668	0.262833	0.085*	
C5	1.2994 (6)	0.4661 (2)	0.2953 (4)	0.0523 (16)	
C6	1.2458 (6)	0.5001 (2)	0.3268 (4)	0.0486 (15)	
C7	1.1397 (5)	0.49365 (17)	0.3512 (3)	0.0402 (13)	
C8	1.0913 (6)	0.52524 (18)	0.3849 (4)	0.0467 (15)	
H8	1.022665	0.522019	0.403556	0.056*	
C9	1.1438 (7)	0.56144 (19)	0.3910 (4)	0.0528 (17)	
H9	1.109226	0.581995	0.413173	0.063*	
C10	1.2451 (8)	0.5672 (2)	0.3650 (4)	0.063 (2)	
H10	1.278324	0.591655	0.368465	0.076*	
C11	1.2982 (7)	0.5366 (2)	0.3334 (5)	0.062 (2)	
H11	1.367952	0.540130	0.316615	0.074*	
C12	1.0618 (7)	0.4635 (2)	0.1662 (4)	0.0530 (16)	
H12	1.137046	0.457694	0.168323	0.064*	
C13	1.0090 (7)	0.4755 (2)	0.1009 (4)	0.0581 (19)	
H13	1.047858	0.477186	0.059557	0.070*	
C14	0.8963 (7)	0.4849 (2)	0.0980 (4)	0.060 (2)	
H14	0.859585	0.493610	0.054856	0.072*	
C15	0.8421 (6)	0.4813 (2)	0.1581 (4)	0.0503 (16)	
H15	0.766948	0.487193	0.156224	0.060*	
C16	0.8964 (6)	0.46876 (17)	0.2241 (4)	0.0437 (14)	
C17	0.8474 (5)	0.46419 (16)	0.2925 (3)	0.0394 (13)	
C18	0.7338 (5)	0.47131 (17)	0.3000 (4)	0.0449 (14)	
H18	0.686257	0.476464	0.259134	0.054*	
C19	0.6941 (6)	0.47065 (19)	0.3666 (4)	0.0491 (16)	
H19	0.619008	0.474812	0.371028	0.059*	
C20	0.7654 (6)	0.46373 (18)	0.4289 (4)	0.0466 (15)	
H20	0.738553	0.464669	0.474737	0.056*	
C21	0.8754 (5)	0.45555 (16)	0.4216 (3)	0.0371 (12)	
H21	0.921882	0.450794	0.463135	0.045*	
C22	0.9199 (5)	0.45412 (15)	0.3538 (3)	0.0335 (11)	
C23	0.9639 (5)	0.36784 (18)	0.2508 (3)	0.0393 (13)	
H23	0.947662	0.385117	0.212643	0.047*	
C24	0.9291 (5)	0.3295 (2)	0.2424 (3)	0.0453 (14)	
H24	0.892515	0.320940	0.199101	0.054*	
C25	0.9501 (6)	0.30434 (18)	0.2999 (4)	0.0450 (14)	
H25	0.927257	0.278454	0.296228	0.054*	
C26	1.0051 (6)	0.31783 (18)	0.3624 (4)	0.0444 (14)	
H26	1.019251	0.301026	0.401604	0.053*	
C27	1.0402 (5)	0.35680 (16)	0.3680 (3)	0.0354 (12)	
C28	1.0981 (5)	0.37445 (16)	0.4324 (3)	0.0350 (12)	
C29	1.1200 (5)	0.41497 (16)	0.4284 (3)	0.0339 (11)	
C30	1.1738 (5)	0.43178 (18)	0.4908 (4)	0.0439 (14)	
H30	1.189190	0.458368	0.490710	0.053*	
C31	1.2051 (6)	0.4101 (2)	0.5529 (4)	0.0497 (16)	
C32	1.1826 (6)	0.3705 (2)	0.5553 (4)	0.0483 (15)	
H32	1.204135	0.355877	0.596429	0.058*	
C33	1.1271 (6)	0.35296 (18)	0.4950 (4)	0.0460 (15)	
H33	1.109168	0.326601	0.496539	0.055*	
C34	1.2727 (7)	0.4294 (2)	0.6147 (4)	0.062 (2)	
H34A	1.350352	0.423451	0.610741	0.075*	
H34B	1.263922	0.457484	0.610190	0.075*	
C35	1.1267 (7)	0.4251 (2)	0.7043 (5)	0.064 (2)	
H35A	1.076578	0.410421	0.670985	0.076*	
H35B	1.110752	0.452614	0.697266	0.076*	
C36	1.1083 (8)	0.4140 (3)	0.7800 (5)	0.073 (2)	
H36A	1.155344	0.429654	0.813559	0.088*	
H36B	1.127931	0.386775	0.787973	0.088*	
C37	0.9843 (8)	0.4204 (3)	0.7947 (5)	0.083 (3)	
H37A	0.966255	0.447789	0.788417	0.100*	
H37B	0.937763	0.405752	0.759177	0.100*	
C38	0.9596 (9)	0.4083 (4)	0.8668 (6)	0.098 (4)	
H38A	0.998248	0.425613	0.901877	0.118*	
H38B	0.988682	0.382255	0.875582	0.118*	
C39	0.8353 (10)	0.4083 (4)	0.8805 (8)	0.112 (4)	
H39A	0.797533	0.390370	0.846225	0.134*	
H39B	0.828054	0.397945	0.928773	0.134*	
C40	0.7766 (12)	0.4461 (5)	0.8747 (9)	0.126 (5)	
H40A	0.787910	0.457969	0.828764	0.189*	
H40B	0.805067	0.463033	0.913256	0.189*	
H40C	0.698808	0.442000	0.878431	0.189*	
C41	0.5498 (6)	0.2789 (2)	0.5490 (4)	0.0499 (16)	
H41	0.574054	0.300487	0.524073	0.060*	
C42	0.4508 (6)	0.2816 (3)	0.5817 (4)	0.0590 (19)	
H42	0.408551	0.304268	0.578837	0.071*	
C43	0.4169 (6)	0.2486 (3)	0.6194 (4)	0.064 (2)	
H43	0.352423	0.249338	0.643691	0.077*	
C44	0.4783 (6)	0.2158 (3)	0.6202 (4)	0.0558 (18)	
H44	0.453430	0.193530	0.642478	0.067*	
C45	0.5781 (5)	0.2149 (2)	0.5882 (3)	0.0441 (15)	
C46	0.6555 (5)	0.18172 (19)	0.5898 (3)	0.0423 (14)	
C47	0.7542 (5)	0.18778 (17)	0.5566 (3)	0.0418 (14)	
C48	0.8281 (6)	0.15588 (18)	0.5553 (4)	0.0473 (15)	
H48	0.894588	0.158848	0.533847	0.057*	
C49	0.8031 (7)	0.1205 (2)	0.5853 (4)	0.0555 (18)	
H49	0.852789	0.099886	0.583297	0.067*	
C50	0.7073 (7)	0.1150 (2)	0.6178 (5)	0.064 (2)	
H50	0.692256	0.090885	0.637860	0.076*	
C51	0.6315 (7)	0.1455 (3)	0.6213 (4)	0.063 (2)	
H51	0.566181	0.141948	0.643890	0.076*	
C52	0.8091 (6)	0.2805 (2)	0.6618 (4)	0.0507 (16)	
H52	0.733148	0.277643	0.664972	0.061*	
C53	0.8708 (7)	0.3000 (2)	0.7166 (4)	0.0605 (19)	
H53	0.837146	0.310114	0.756032	0.073*	
C54	0.9818 (7)	0.3040 (2)	0.7116 (4)	0.066 (2)	
H54	1.024160	0.317916	0.747001	0.079*	
C55	1.0308 (6)	0.2878 (2)	0.6553 (4)	0.057 (2)	
H55	1.107096	0.289922	0.653051	0.068*	
C56	0.9670 (5)	0.26793 (18)	0.6006 (4)	0.0444 (15)	
C57	1.0098 (5)	0.24927 (19)	0.5357 (4)	0.0465 (15)	
C58	0.9277 (5)	0.23316 (17)	0.4857 (4)	0.0409 (13)	
C59	0.9663 (6)	0.2147 (2)	0.4258 (4)	0.0491 (15)	
H59	0.915836	0.203917	0.390780	0.059*	
C60	1.0815 (7)	0.2119 (2)	0.4170 (5)	0.063 (2)	
H60	1.105394	0.199287	0.376449	0.075*	
C61	1.1573 (7)	0.2274 (3)	0.4669 (6)	0.070 (2)	
H61	1.232616	0.225364	0.460704	0.084*	
C62	1.1224 (7)	0.2460 (2)	0.5260 (5)	0.065 (2)	
H62	1.174177	0.256581	0.560357	0.078*	
C63	0.7952 (6)	0.32935 (19)	0.4802 (4)	0.0491 (15)	
H63	0.831443	0.330229	0.526458	0.059*	
C64	0.7811 (6)	0.36363 (19)	0.4425 (4)	0.0530 (17)	
H64	0.805441	0.387149	0.463244	0.064*	
C65	0.7303 (7)	0.3625 (2)	0.3734 (4)	0.0555 (18)	
H65	0.721146	0.385217	0.346211	0.067*	
C66	0.6933 (5)	0.32747 (19)	0.3450 (4)	0.0455 (14)	
H66	0.657922	0.326505	0.298429	0.055*	
C67	0.7084 (5)	0.29345 (17)	0.3854 (3)	0.0368 (12)	
C68	0.6741 (5)	0.25362 (17)	0.3615 (3)	0.0343 (11)	
C69	0.6989 (5)	0.22348 (17)	0.4136 (3)	0.0356 (12)	
C70	0.6660 (5)	0.18595 (17)	0.3923 (3)	0.0375 (12)	
H70	0.684364	0.165287	0.423547	0.045*	
C71	0.6078 (5)	0.17814 (18)	0.3272 (4)	0.0423 (13)	
C72	0.5840 (6)	0.2087 (2)	0.2768 (4)	0.0494 (16)	
H72	0.545540	0.203752	0.232143	0.059*	
C73	0.6187 (6)	0.24592 (19)	0.2951 (4)	0.0462 (15)	
H73	0.604543	0.266161	0.262121	0.055*	
C74	0.5677 (6)	0.1377 (2)	0.3095 (4)	0.0498 (16)	
H74A	0.564341	0.134023	0.257347	0.060*	
H74B	0.620452	0.119107	0.331592	0.060*	
C75	0.4457 (7)	0.1345 (3)	0.4145 (5)	0.067 (2)	
H75A	0.459328	0.161505	0.428261	0.081*	
H75B	0.500241	0.118398	0.441611	0.081*	
C76	0.3303 (9)	0.1228 (3)	0.4325 (5)	0.083 (3)	
H76A	0.324515	0.094587	0.428812	0.100*	0.412 (13)
H76B	0.277466	0.133770	0.395918	0.100*	0.412 (13)
H76C	0.306827	0.099206	0.406568	0.100*	0.588 (13)
H76D	0.277521	0.143371	0.419445	0.100*	0.588 (13)
C77	0.2953 (14)	0.1351 (5)	0.5082 (8)	0.054 (3)	0.412 (13)
H77A	0.347486	0.124352	0.545518	0.065*	0.412 (13)
H77B	0.297884	0.163272	0.512146	0.065*	0.412 (13)
C77A	0.3386 (16)	0.1154 (7)	0.5185 (8)	0.090 (4)	0.588 (13)
H77C	0.405021	0.100376	0.532153	0.108*	0.588 (13)
H77D	0.345233	0.140260	0.543466	0.108*	0.588 (13)
C78	0.1840 (16)	0.1215 (7)	0.5202 (10)	0.067 (3)	0.412 (13)
H78A	0.177213	0.094476	0.505158	0.080*	0.412 (13)
H78B	0.131041	0.136550	0.489450	0.080*	0.412 (13)
C78A	0.2409 (14)	0.0942 (6)	0.5424 (13)	0.102 (4)	0.588 (13)
H78C	0.259008	0.083001	0.590117	0.122*	0.588 (13)
H78D	0.221646	0.072989	0.508917	0.122*	0.588 (13)
C79	0.1531 (18)	0.1248 (6)	0.5994 (11)	0.067 (3)	0.412 (13)
H79A	0.076702	0.116498	0.601059	0.080*	0.412 (13)
H79B	0.198849	0.106574	0.628620	0.080*	0.412 (13)
C79A	0.1412 (16)	0.1218 (6)	0.5454 (12)	0.099 (3)	0.588 (13)
H79C	0.131299	0.135653	0.499502	0.119*	0.588 (13)
H79D	0.075296	0.106112	0.549784	0.119*	0.588 (13)
C80	0.1650 (19)	0.1640 (6)	0.6337 (14)	0.068 (4)	0.412 (13)
H80A	0.106664	0.180731	0.613881	0.102*	0.412 (13)
H80B	0.235434	0.174945	0.624230	0.102*	0.412 (13)
H80C	0.160575	0.161567	0.685198	0.102*	0.412 (13)
C80A	0.149 (2)	0.1505 (7)	0.6040 (12)	0.102 (4)	0.588 (13)
H80D	0.092408	0.170046	0.594490	0.153*	0.588 (13)
H80E	0.220302	0.162577	0.606797	0.153*	0.588 (13)
H80F	0.137666	0.137751	0.649155	0.153*	0.588 (13)

### Atomic displacement parameters (Å^2^)

**Table:**

	*U*^11^	*U*^22^	*U*^33^	*U*^12^	*U*^13^	*U*^23^
Ir1	0.03986 (14)	0.03253 (13)	0.03194 (13)	−0.00881 (9)	−0.00058 (10)	0.00123 (9)
Ir2	0.03531 (13)	0.03230 (13)	0.03245 (13)	−0.00578 (9)	−0.00788 (9)	0.00281 (9)
Cl1	0.0552 (9)	0.0412 (8)	0.0675 (11)	0.0030 (7)	−0.0152 (8)	−0.0086 (7)
Cl2	0.0564 (9)	0.0473 (8)	0.0701 (11)	−0.0028 (7)	−0.0264 (8)	0.0008 (8)
N1	0.041 (3)	0.052 (3)	0.039 (3)	−0.011 (2)	0.005 (2)	−0.001 (2)
N2	0.054 (3)	0.034 (2)	0.033 (3)	−0.011 (2)	−0.001 (2)	0.003 (2)
N3	0.037 (2)	0.032 (2)	0.033 (2)	−0.0083 (19)	0.0011 (19)	−0.0016 (19)
N4	0.061 (4)	0.048 (3)	0.052 (3)	−0.002 (3)	−0.017 (3)	0.001 (3)
N5	0.036 (2)	0.051 (3)	0.026 (2)	−0.002 (2)	−0.0002 (19)	0.000 (2)
N6	0.048 (3)	0.042 (3)	0.035 (3)	−0.012 (2)	−0.016 (2)	0.001 (2)
N7	0.035 (2)	0.035 (2)	0.041 (3)	−0.0025 (19)	0.000 (2)	0.000 (2)
N8	0.054 (3)	0.044 (3)	0.058 (4)	−0.004 (2)	−0.015 (3)	−0.008 (3)
C1	0.051 (4)	0.062 (4)	0.064 (5)	−0.014 (3)	0.009 (3)	−0.008 (4)
C2	0.055 (5)	0.090 (7)	0.093 (7)	−0.007 (4)	0.024 (5)	−0.018 (5)
C3	0.055 (5)	0.103 (8)	0.096 (7)	−0.020 (5)	0.019 (5)	−0.020 (6)
C4	0.054 (4)	0.089 (6)	0.070 (5)	−0.030 (4)	0.011 (4)	−0.003 (5)
C5	0.047 (4)	0.059 (4)	0.050 (4)	−0.017 (3)	−0.002 (3)	0.005 (3)
C6	0.057 (4)	0.051 (4)	0.038 (3)	−0.020 (3)	0.002 (3)	0.005 (3)
C7	0.049 (3)	0.037 (3)	0.033 (3)	−0.014 (3)	−0.005 (2)	0.004 (2)
C8	0.062 (4)	0.038 (3)	0.039 (3)	−0.009 (3)	−0.005 (3)	0.003 (3)
C9	0.081 (5)	0.038 (3)	0.036 (3)	−0.008 (3)	−0.009 (3)	−0.001 (3)
C10	0.086 (6)	0.047 (4)	0.056 (4)	−0.030 (4)	−0.002 (4)	0.008 (3)
C11	0.060 (4)	0.061 (5)	0.063 (5)	−0.025 (4)	0.002 (4)	0.010 (4)
C12	0.065 (4)	0.047 (4)	0.049 (4)	−0.008 (3)	0.009 (3)	0.008 (3)
C13	0.078 (5)	0.058 (4)	0.040 (4)	−0.009 (4)	0.018 (3)	0.008 (3)
C14	0.087 (6)	0.051 (4)	0.039 (4)	−0.010 (4)	−0.013 (4)	0.007 (3)
C15	0.059 (4)	0.043 (3)	0.047 (4)	−0.006 (3)	−0.007 (3)	0.003 (3)
C16	0.057 (4)	0.028 (3)	0.044 (3)	−0.009 (3)	−0.007 (3)	0.001 (2)
C17	0.046 (3)	0.028 (3)	0.043 (3)	−0.007 (2)	−0.008 (3)	0.002 (2)
C18	0.043 (3)	0.032 (3)	0.058 (4)	−0.002 (2)	−0.009 (3)	0.007 (3)
C19	0.040 (3)	0.038 (3)	0.068 (5)	0.000 (3)	0.000 (3)	0.000 (3)
C20	0.050 (4)	0.034 (3)	0.056 (4)	−0.002 (3)	0.007 (3)	−0.003 (3)
C21	0.041 (3)	0.031 (3)	0.039 (3)	−0.005 (2)	−0.002 (2)	−0.001 (2)
C22	0.039 (3)	0.023 (2)	0.038 (3)	−0.007 (2)	−0.002 (2)	0.000 (2)
C23	0.042 (3)	0.040 (3)	0.036 (3)	−0.007 (2)	0.001 (2)	0.000 (2)
C24	0.048 (3)	0.050 (4)	0.037 (3)	−0.009 (3)	−0.004 (3)	−0.006 (3)
C25	0.058 (4)	0.034 (3)	0.042 (3)	−0.007 (3)	−0.006 (3)	−0.002 (2)
C26	0.052 (4)	0.034 (3)	0.047 (4)	−0.004 (3)	0.001 (3)	−0.002 (3)
C27	0.036 (3)	0.033 (3)	0.037 (3)	−0.006 (2)	0.002 (2)	−0.002 (2)
C28	0.040 (3)	0.035 (3)	0.029 (3)	−0.005 (2)	−0.003 (2)	0.004 (2)
C29	0.039 (3)	0.034 (3)	0.027 (3)	0.000 (2)	−0.004 (2)	0.003 (2)
C30	0.047 (3)	0.035 (3)	0.049 (4)	−0.011 (3)	−0.005 (3)	0.002 (3)
C31	0.061 (4)	0.045 (3)	0.040 (3)	−0.011 (3)	−0.014 (3)	0.005 (3)
C32	0.058 (4)	0.045 (3)	0.039 (3)	−0.006 (3)	−0.013 (3)	0.004 (3)
C33	0.060 (4)	0.032 (3)	0.045 (3)	−0.009 (3)	−0.003 (3)	0.002 (3)
C34	0.078 (5)	0.056 (4)	0.050 (4)	−0.025 (4)	−0.014 (4)	0.006 (3)
C35	0.060 (5)	0.060 (5)	0.068 (5)	0.004 (4)	−0.017 (4)	−0.013 (4)
C36	0.083 (6)	0.066 (5)	0.069 (6)	−0.002 (4)	−0.003 (5)	−0.017 (4)
C37	0.075 (6)	0.099 (7)	0.072 (6)	−0.024 (5)	−0.019 (5)	0.008 (5)
C38	0.088 (7)	0.106 (8)	0.097 (8)	−0.027 (6)	−0.011 (6)	0.029 (7)
C39	0.099 (9)	0.126 (11)	0.109 (10)	−0.030 (8)	0.005 (7)	0.023 (8)
C40	0.107 (10)	0.143 (13)	0.125 (11)	−0.061 (10)	−0.010 (8)	0.011 (10)
C41	0.042 (3)	0.068 (4)	0.039 (3)	−0.003 (3)	−0.005 (3)	−0.005 (3)
C42	0.041 (4)	0.088 (6)	0.047 (4)	0.012 (4)	−0.002 (3)	−0.005 (4)
C43	0.038 (4)	0.114 (7)	0.039 (4)	−0.010 (4)	0.000 (3)	0.000 (4)
C44	0.044 (4)	0.082 (5)	0.039 (3)	−0.008 (4)	−0.010 (3)	0.002 (3)
C45	0.040 (3)	0.067 (4)	0.025 (3)	−0.019 (3)	−0.006 (2)	0.003 (3)
C46	0.049 (3)	0.049 (3)	0.027 (3)	−0.016 (3)	−0.007 (2)	0.009 (2)
C47	0.050 (3)	0.039 (3)	0.033 (3)	−0.017 (3)	−0.021 (3)	0.009 (2)
C48	0.058 (4)	0.038 (3)	0.043 (3)	−0.002 (3)	−0.015 (3)	0.007 (3)
C49	0.070 (5)	0.044 (4)	0.049 (4)	−0.001 (3)	−0.020 (3)	0.008 (3)
C50	0.073 (5)	0.051 (4)	0.065 (5)	−0.010 (4)	−0.005 (4)	0.023 (4)
C51	0.066 (5)	0.077 (5)	0.046 (4)	−0.027 (4)	−0.007 (3)	0.018 (4)
C52	0.058 (4)	0.054 (4)	0.038 (3)	−0.011 (3)	−0.008 (3)	0.003 (3)
C53	0.073 (5)	0.061 (4)	0.045 (4)	−0.013 (4)	−0.010 (4)	−0.005 (3)
C54	0.078 (5)	0.064 (5)	0.050 (4)	−0.032 (4)	−0.024 (4)	0.001 (4)
C55	0.058 (4)	0.053 (4)	0.054 (4)	−0.021 (3)	−0.026 (3)	0.015 (3)
C56	0.047 (3)	0.039 (3)	0.044 (3)	−0.013 (3)	−0.016 (3)	0.009 (3)
C57	0.033 (3)	0.042 (3)	0.062 (4)	−0.004 (2)	−0.011 (3)	0.017 (3)
C58	0.040 (3)	0.035 (3)	0.046 (3)	0.001 (2)	−0.006 (3)	0.007 (3)
C59	0.045 (3)	0.044 (3)	0.058 (4)	0.001 (3)	−0.001 (3)	0.001 (3)
C60	0.053 (4)	0.060 (4)	0.077 (5)	0.014 (3)	0.015 (4)	0.002 (4)
C61	0.044 (4)	0.062 (5)	0.102 (7)	0.007 (4)	−0.002 (4)	0.013 (5)
C62	0.050 (4)	0.052 (4)	0.089 (6)	−0.006 (3)	−0.021 (4)	0.015 (4)
C63	0.055 (4)	0.041 (3)	0.050 (4)	−0.011 (3)	−0.004 (3)	−0.001 (3)
C64	0.060 (4)	0.035 (3)	0.063 (4)	−0.005 (3)	0.000 (3)	0.000 (3)
C65	0.069 (5)	0.036 (3)	0.063 (5)	0.002 (3)	0.014 (4)	0.016 (3)
C66	0.047 (3)	0.042 (3)	0.046 (3)	−0.001 (3)	−0.001 (3)	0.012 (3)
C67	0.036 (3)	0.036 (3)	0.038 (3)	0.000 (2)	−0.003 (2)	0.005 (2)
C68	0.037 (3)	0.037 (3)	0.027 (3)	−0.001 (2)	−0.001 (2)	0.004 (2)
C69	0.034 (3)	0.036 (3)	0.035 (3)	0.001 (2)	−0.009 (2)	0.007 (2)
C70	0.036 (3)	0.035 (3)	0.040 (3)	0.001 (2)	−0.009 (2)	0.003 (2)
C71	0.041 (3)	0.040 (3)	0.044 (3)	−0.002 (2)	−0.008 (3)	−0.008 (3)
C72	0.052 (4)	0.058 (4)	0.036 (3)	0.001 (3)	−0.016 (3)	−0.001 (3)
C73	0.053 (4)	0.045 (3)	0.040 (3)	0.006 (3)	−0.007 (3)	0.008 (3)
C74	0.047 (4)	0.047 (4)	0.054 (4)	0.001 (3)	−0.004 (3)	−0.015 (3)
C75	0.074 (5)	0.063 (5)	0.062 (5)	−0.021 (4)	−0.007 (4)	−0.014 (4)
C76	0.082 (6)	0.085 (6)	0.083 (6)	−0.033 (5)	0.005 (5)	−0.019 (5)
C77	0.061 (4)	0.036 (7)	0.066 (5)	0.016 (4)	0.000 (4)	0.023 (5)
C77A	0.089 (4)	0.110 (7)	0.071 (9)	−0.010 (4)	0.009 (5)	−0.012 (7)
C78	0.065 (4)	0.066 (6)	0.069 (4)	0.005 (5)	0.002 (4)	0.020 (4)
C78A	0.087 (4)	0.117 (6)	0.103 (9)	−0.005 (4)	0.011 (5)	0.009 (5)
C79	0.062 (6)	0.069 (5)	0.069 (4)	0.002 (5)	0.001 (4)	0.018 (4)
C79A	0.092 (4)	0.122 (6)	0.085 (7)	0.001 (4)	0.017 (5)	0.018 (5)
C80	0.062 (11)	0.070 (5)	0.072 (5)	−0.001 (5)	0.006 (6)	0.016 (4)
C80A	0.105 (9)	0.119 (7)	0.082 (7)	0.011 (7)	0.007 (6)	0.021 (5)

### Geometric parameters (Å, º)

**Table:**

Ir1—N1	2.132 (5)	C38—H38B	0.9700
Ir1—N2	2.131 (5)	C38—C39	1.544 (14)
Ir1—N3	2.144 (4)	C39—H39A	0.9700
Ir1—C7	2.024 (6)	C39—H39B	0.9700
Ir1—C22	2.015 (6)	C39—C40	1.478 (19)
Ir1—C29	2.036 (5)	C40—H40A	0.9600
Ir2—N5	2.125 (5)	C40—H40B	0.9600
Ir2—N6	2.119 (5)	C40—H40C	0.9600
Ir2—N7	2.105 (5)	C41—H41	0.9300
Ir2—C47	2.028 (6)	C41—C42	1.387 (10)
Ir2—C58	2.013 (6)	C42—H42	0.9300
Ir2—C69	2.010 (6)	C42—C43	1.407 (12)
N1—C1	1.315 (10)	C43—H43	0.9300
N1—C5	1.373 (8)	C43—C44	1.344 (12)
N2—C12	1.354 (9)	C44—H44	0.9300
N2—C16	1.371 (9)	C44—C45	1.384 (10)
N3—C23	1.332 (7)	C45—C46	1.471 (10)
N3—C27	1.355 (7)	C46—C47	1.399 (9)
N4—H4A	0.8900	C46—C51	1.412 (10)
N4—H4B	0.8900	C47—C48	1.414 (10)
N4—C34	1.503 (10)	C48—H48	0.9300
N4—C35	1.469 (10)	C48—C49	1.377 (9)
N5—C41	1.334 (9)	C49—H49	0.9300
N5—C45	1.362 (8)	C49—C50	1.360 (12)
N6—C52	1.347 (9)	C50—H50	0.9300
N6—C56	1.359 (9)	C50—C51	1.394 (13)
N7—C63	1.344 (8)	C51—H51	0.9300
N7—C67	1.372 (8)	C52—H52	0.9300
N8—H8A	0.8900	C52—C53	1.382 (10)
N8—H8B	0.8900	C53—H53	0.9300
N8—C74	1.488 (9)	C53—C54	1.360 (12)
N8—C75	1.485 (10)	C54—H54	0.9300
C1—H1	0.9300	C54—C55	1.355 (13)
C1—C2	1.402 (11)	C55—H55	0.9300
C2—H2	0.9300	C55—C56	1.401 (9)
C2—C3	1.386 (14)	C56—C57	1.486 (11)
C3—H3	0.9300	C57—C58	1.414 (9)
C3—C4	1.358 (14)	C57—C62	1.393 (11)
C4—H4	0.9300	C58—C59	1.388 (10)
C4—C5	1.386 (11)	C59—H59	0.9300
C5—C6	1.473 (11)	C59—C60	1.419 (10)
C6—C7	1.411 (9)	C60—H60	0.9300
C6—C11	1.403 (10)	C60—C61	1.357 (13)
C7—C8	1.399 (10)	C61—H61	0.9300
C8—H8	0.9300	C61—C62	1.359 (14)
C8—C9	1.394 (9)	C62—H62	0.9300
C9—H9	0.9300	C63—H63	0.9300
C9—C10	1.366 (12)	C63—C64	1.369 (10)
C10—H10	0.9300	C64—H64	0.9300
C10—C11	1.383 (13)	C64—C65	1.373 (11)
C11—H11	0.9300	C65—H65	0.9300
C12—H12	0.9300	C65—C66	1.371 (10)
C12—C13	1.381 (11)	C66—H66	0.9300
C13—H13	0.9300	C66—C67	1.389 (8)
C13—C14	1.398 (12)	C67—C68	1.483 (8)
C14—H14	0.9300	C68—C69	1.428 (8)
C14—C15	1.340 (11)	C68—C73	1.377 (9)
C15—H15	0.9300	C69—C70	1.394 (8)
C15—C16	1.406 (9)	C70—H70	0.9300
C16—C17	1.445 (9)	C70—C71	1.371 (8)
C17—C18	1.413 (9)	C71—C72	1.416 (9)
C17—C22	1.419 (8)	C71—C74	1.495 (9)
C18—H18	0.9300	C72—H72	0.9300
C18—C19	1.355 (10)	C72—C73	1.376 (10)
C19—H19	0.9300	C73—H73	0.9300
C19—C20	1.403 (10)	C74—H74A	0.9700
C20—H20	0.9300	C74—H74B	0.9700
C20—C21	1.378 (9)	C75—H75A	0.9700
C21—H21	0.9300	C75—H75B	0.9700
C21—C22	1.401 (8)	C75—C76	1.515 (13)
C23—H23	0.9300	C76—H76A	0.9700
C23—C24	1.386 (9)	C76—H76B	0.9700
C24—H24	0.9300	C76—H76C	0.9700
C24—C25	1.375 (9)	C76—H76D	0.9700
C25—H25	0.9300	C76—C77	1.549 (15)
C25—C26	1.368 (9)	C76—C77A	1.604 (15)
C26—H26	0.9300	C77—H77A	0.9700
C26—C27	1.403 (8)	C77—H77B	0.9700
C27—C28	1.463 (8)	C77—C78	1.457 (17)
C28—C29	1.417 (8)	C77A—H77C	0.9700
C28—C33	1.393 (8)	C77A—H77D	0.9700
C29—C30	1.402 (8)	C77A—C78A	1.484 (17)
C30—H30	0.9300	C78—H78A	0.9700
C30—C31	1.394 (9)	C78—H78B	0.9700
C31—C32	1.386 (9)	C78—C79	1.542 (18)
C31—C34	1.506 (9)	C78A—H78C	0.9700
C32—H32	0.9300	C78A—H78D	0.9700
C32—C33	1.390 (9)	C78A—C79A	1.538 (18)
C33—H33	0.9300	C79—H79A	0.9700
C34—H34A	0.9700	C79—H79B	0.9700
C34—H34B	0.9700	C79—C80	1.49 (3)
C35—H35A	0.9700	C79A—H79C	0.9700
C35—H35B	0.9700	C79A—H79D	0.9700
C35—C36	1.482 (13)	C79A—C80A	1.46 (3)
C36—H36A	0.9700	C80—H80A	0.9600
C36—H36B	0.9700	C80—H80B	0.9600
C36—C37	1.561 (12)	C80—H80C	0.9600
C37—H37A	0.9700	C80A—H80D	0.9600
C37—H37B	0.9700	C80A—H80E	0.9600
C37—C38	1.447 (12)	C80A—H80F	0.9600
C38—H38A	0.9700		
			
N1—Ir1—N3	96.9 (2)	H38A—C38—H38B	107.5
N2—Ir1—N1	93.9 (2)	C39—C38—H38A	108.5
N2—Ir1—N3	93.27 (18)	C39—C38—H38B	108.5
C7—Ir1—N1	79.1 (2)	C38—C39—H39A	108.0
C7—Ir1—N2	89.6 (2)	C38—C39—H39B	108.0
C7—Ir1—N3	175.3 (2)	H39A—C39—H39B	107.3
C7—Ir1—C29	98.1 (2)	C40—C39—C38	117.1 (11)
C22—Ir1—N1	171.5 (2)	C40—C39—H39A	108.0
C22—Ir1—N2	79.4 (2)	C40—C39—H39B	108.0
C22—Ir1—N3	88.74 (19)	C39—C40—H40A	109.5
C22—Ir1—C7	95.5 (2)	C39—C40—H40B	109.5
C22—Ir1—C29	95.9 (2)	C39—C40—H40C	109.5
C29—Ir1—N1	91.4 (2)	H40A—C40—H40B	109.5
C29—Ir1—N2	171.3 (2)	H40A—C40—H40C	109.5
C29—Ir1—N3	79.3 (2)	H40B—C40—H40C	109.5
N6—Ir2—N5	93.7 (2)	N5—C41—H41	118.6
N7—Ir2—N5	94.34 (19)	N5—C41—C42	122.7 (7)
N7—Ir2—N6	93.57 (19)	C42—C41—H41	118.6
C47—Ir2—N5	79.3 (2)	C41—C42—H42	121.4
C47—Ir2—N6	93.8 (2)	C41—C42—C43	117.3 (8)
C47—Ir2—N7	170.5 (2)	C43—C42—H42	121.4
C58—Ir2—N5	171.6 (2)	C42—C43—H43	120.2
C58—Ir2—N6	79.9 (2)	C44—C43—C42	119.5 (7)
C58—Ir2—N7	91.5 (2)	C44—C43—H43	120.2
C58—Ir2—C47	95.6 (3)	C43—C44—H44	119.5
C69—Ir2—N5	90.7 (2)	C43—C44—C45	121.1 (8)
C69—Ir2—N6	172.1 (2)	C45—C44—H44	119.5
C69—Ir2—N7	79.6 (2)	N5—C45—C44	119.8 (7)
C69—Ir2—C47	93.4 (2)	N5—C45—C46	114.3 (5)
C69—Ir2—C58	96.3 (3)	C44—C45—C46	125.9 (6)
C1—N1—Ir1	126.4 (5)	C47—C46—C45	116.2 (5)
C1—N1—C5	118.7 (6)	C47—C46—C51	121.4 (7)
C5—N1—Ir1	114.8 (5)	C51—C46—C45	122.4 (6)
C12—N2—Ir1	126.6 (5)	C46—C47—Ir2	115.5 (5)
C12—N2—C16	119.0 (6)	C46—C47—C48	117.1 (6)
C16—N2—Ir1	114.4 (4)	C48—C47—Ir2	127.3 (5)
C23—N3—Ir1	126.5 (4)	C47—C48—H48	119.5
C23—N3—C27	119.1 (5)	C49—C48—C47	121.0 (7)
C27—N3—Ir1	114.3 (4)	C49—C48—H48	119.5
H4A—N4—H4B	107.4	C48—C49—H49	119.3
C34—N4—H4A	108.3	C50—C49—C48	121.4 (7)
C34—N4—H4B	108.3	C50—C49—H49	119.3
C35—N4—H4A	108.3	C49—C50—H50	119.9
C35—N4—H4B	108.3	C49—C50—C51	120.3 (7)
C35—N4—C34	115.8 (6)	C51—C50—H50	119.9
C41—N5—Ir2	125.6 (5)	C46—C51—H51	120.6
C41—N5—C45	119.5 (6)	C50—C51—C46	118.8 (7)
C45—N5—Ir2	114.7 (4)	C50—C51—H51	120.6
C52—N6—Ir2	126.4 (5)	N6—C52—H52	119.0
C52—N6—C56	119.2 (5)	N6—C52—C53	122.0 (7)
C56—N6—Ir2	114.2 (4)	C53—C52—H52	119.0
C63—N7—Ir2	126.5 (4)	C52—C53—H53	120.6
C63—N7—C67	117.5 (5)	C54—C53—C52	118.7 (8)
C67—N7—Ir2	115.9 (4)	C54—C53—H53	120.6
H8A—N8—H8B	107.3	C53—C54—H54	119.9
C74—N8—H8A	108.2	C55—C54—C53	120.2 (7)
C74—N8—H8B	108.2	C55—C54—H54	119.9
C75—N8—H8A	108.2	C54—C55—H55	119.9
C75—N8—H8B	108.2	C54—C55—C56	120.2 (7)
C75—N8—C74	116.5 (6)	C56—C55—H55	119.9
N1—C1—H1	117.9	N6—C56—C55	119.5 (7)
N1—C1—C2	124.3 (8)	N6—C56—C57	114.8 (5)
C2—C1—H1	117.9	C55—C56—C57	125.7 (7)
C1—C2—H2	121.9	C58—C57—C56	115.0 (6)
C3—C2—C1	116.1 (9)	C62—C57—C56	123.0 (7)
C3—C2—H2	121.9	C62—C57—C58	121.9 (8)
C2—C3—H3	119.7	C57—C58—Ir2	115.6 (5)
C4—C3—C2	120.6 (9)	C59—C58—Ir2	128.6 (5)
C4—C3—H3	119.7	C59—C58—C57	115.9 (6)
C3—C4—H4	119.8	C58—C59—H59	119.5
C3—C4—C5	120.4 (8)	C58—C59—C60	121.1 (7)
C5—C4—H4	119.8	C60—C59—H59	119.5
N1—C5—C4	119.8 (7)	C59—C60—H60	119.5
N1—C5—C6	113.6 (6)	C61—C60—C59	120.9 (8)
C4—C5—C6	126.6 (7)	C61—C60—H60	119.5
C7—C6—C5	116.3 (6)	C60—C61—H61	120.2
C11—C6—C5	121.9 (7)	C60—C61—C62	119.5 (8)
C11—C6—C7	121.8 (7)	C62—C61—H61	120.2
C6—C7—Ir1	115.5 (5)	C57—C62—H62	119.7
C8—C7—Ir1	128.1 (5)	C61—C62—C57	120.6 (8)
C8—C7—C6	116.4 (6)	C61—C62—H62	119.7
C7—C8—H8	119.3	N7—C63—H63	118.0
C9—C8—C7	121.4 (7)	N7—C63—C64	123.9 (6)
C9—C8—H8	119.3	C64—C63—H63	118.0
C8—C9—H9	119.5	C63—C64—H64	120.8
C10—C9—C8	121.0 (7)	C63—C64—C65	118.4 (6)
C10—C9—H9	119.5	C65—C64—H64	120.8
C9—C10—H10	120.1	C64—C65—H65	120.3
C9—C10—C11	119.9 (7)	C66—C65—C64	119.5 (6)
C11—C10—H10	120.1	C66—C65—H65	120.3
C6—C11—H11	120.3	C65—C66—H66	119.9
C10—C11—C6	119.4 (7)	C65—C66—C67	120.1 (6)
C10—C11—H11	120.3	C67—C66—H66	119.9
N2—C12—H12	119.0	N7—C67—C66	120.5 (6)
N2—C12—C13	122.0 (7)	N7—C67—C68	113.3 (5)
C13—C12—H12	119.0	C66—C67—C68	126.1 (6)
C12—C13—H13	120.4	C69—C68—C67	115.2 (5)
C12—C13—C14	119.1 (7)	C73—C68—C67	123.2 (5)
C14—C13—H13	120.4	C73—C68—C69	121.6 (6)
C13—C14—H14	120.5	C68—C69—Ir2	115.8 (4)
C15—C14—C13	119.0 (7)	C70—C69—Ir2	128.2 (4)
C15—C14—H14	120.5	C70—C69—C68	115.9 (5)
C14—C15—H15	119.3	C69—C70—H70	118.5
C14—C15—C16	121.5 (7)	C71—C70—C69	123.0 (5)
C16—C15—H15	119.3	C71—C70—H70	118.5
N2—C16—C15	119.4 (6)	C70—C71—C72	119.7 (6)
N2—C16—C17	114.0 (5)	C70—C71—C74	120.5 (6)
C15—C16—C17	126.5 (7)	C72—C71—C74	119.7 (6)
C18—C17—C16	122.4 (6)	C71—C72—H72	120.6
C18—C17—C22	120.6 (6)	C73—C72—C71	118.7 (6)
C22—C17—C16	117.0 (6)	C73—C72—H72	120.6
C17—C18—H18	119.9	C68—C73—H73	119.5
C19—C18—C17	120.2 (6)	C72—C73—C68	121.0 (6)
C19—C18—H18	119.9	C72—C73—H73	119.5
C18—C19—H19	119.7	N8—C74—C71	112.8 (6)
C18—C19—C20	120.6 (6)	N8—C74—H74A	109.0
C20—C19—H19	119.7	N8—C74—H74B	109.0
C19—C20—H20	120.3	C71—C74—H74A	109.0
C21—C20—C19	119.3 (7)	C71—C74—H74B	109.0
C21—C20—H20	120.3	H74A—C74—H74B	107.8
C20—C21—H21	118.8	N8—C75—H75A	109.7
C20—C21—C22	122.5 (6)	N8—C75—H75B	109.7
C22—C21—H21	118.8	N8—C75—C76	109.9 (6)
C17—C22—Ir1	114.6 (4)	H75A—C75—H75B	108.2
C21—C22—Ir1	128.9 (4)	C76—C75—H75A	109.7
C21—C22—C17	116.6 (5)	C76—C75—H75B	109.7
N3—C23—H23	118.3	C75—C76—H76A	108.1
N3—C23—C24	123.3 (6)	C75—C76—H76B	108.1
C24—C23—H23	118.3	C75—C76—H76C	110.6
C23—C24—H24	121.0	C75—C76—H76D	110.6
C25—C24—C23	118.1 (6)	C75—C76—C77	116.6 (9)
C25—C24—H24	121.0	C75—C76—C77A	105.8 (9)
C24—C25—H25	120.4	H76A—C76—H76B	107.3
C26—C25—C24	119.3 (6)	H76C—C76—H76D	108.7
C26—C25—H25	120.4	C77—C76—H76A	108.1
C25—C26—H26	119.7	C77—C76—H76B	108.1
C25—C26—C27	120.5 (6)	C77A—C76—H76C	110.6
C27—C26—H26	119.7	C77A—C76—H76D	110.6
N3—C27—C26	119.6 (5)	C76—C77—H77A	109.3
N3—C27—C28	115.2 (5)	C76—C77—H77B	109.3
C26—C27—C28	125.2 (5)	H77A—C77—H77B	107.9
C29—C28—C27	116.3 (5)	C78—C77—C76	111.8 (14)
C33—C28—C27	122.0 (5)	C78—C77—H77A	109.3
C33—C28—C29	121.7 (5)	C78—C77—H77B	109.3
C28—C29—Ir1	114.9 (4)	C76—C77A—H77C	109.1
C30—C29—Ir1	129.2 (4)	C76—C77A—H77D	109.1
C30—C29—C28	115.9 (5)	H77C—C77A—H77D	107.9
C29—C30—H30	118.8	C78A—C77A—C76	112.4 (14)
C31—C30—C29	122.4 (6)	C78A—C77A—H77C	109.1
C31—C30—H30	118.8	C78A—C77A—H77D	109.1
C30—C31—C34	119.1 (6)	C77—C78—H78A	108.6
C32—C31—C30	120.4 (6)	C77—C78—H78B	108.6
C32—C31—C34	120.2 (6)	C77—C78—C79	114.7 (17)
C31—C32—H32	120.6	H78A—C78—H78B	107.6
C31—C32—C33	118.8 (6)	C79—C78—H78A	108.6
C33—C32—H32	120.6	C79—C78—H78B	108.6
C28—C33—H33	119.6	C77A—C78A—H78C	109.5
C32—C33—C28	120.8 (6)	C77A—C78A—H78D	109.5
C32—C33—H33	119.6	C77A—C78A—C79A	110.8 (18)
N4—C34—C31	114.0 (6)	H78C—C78A—H78D	108.1
N4—C34—H34A	108.8	C79A—C78A—H78C	109.5
N4—C34—H34B	108.8	C79A—C78A—H78D	109.5
C31—C34—H34A	108.8	C78—C79—H79A	108.2
C31—C34—H34B	108.8	C78—C79—H79B	108.2
H34A—C34—H34B	107.7	H79A—C79—H79B	107.3
N4—C35—H35A	109.5	C80—C79—C78	116.5 (18)
N4—C35—H35B	109.5	C80—C79—H79A	108.2
N4—C35—C36	110.7 (7)	C80—C79—H79B	108.2
H35A—C35—H35B	108.1	C78A—C79A—H79C	108.3
C36—C35—H35A	109.5	C78A—C79A—H79D	108.3
C36—C35—H35B	109.5	H79C—C79A—H79D	107.4
C35—C36—H36A	109.6	C80A—C79A—C78A	116 (2)
C35—C36—H36B	109.6	C80A—C79A—H79C	108.3
C35—C36—C37	110.2 (8)	C80A—C79A—H79D	108.3
H36A—C36—H36B	108.1	C79—C80—H80A	109.5
C37—C36—H36A	109.6	C79—C80—H80B	109.5
C37—C36—H36B	109.6	C79—C80—H80C	109.5
C36—C37—H37A	109.0	H80A—C80—H80B	109.5
C36—C37—H37B	109.0	H80A—C80—H80C	109.5
H37A—C37—H37B	107.8	H80B—C80—H80C	109.5
C38—C37—C36	113.0 (8)	C79A—C80A—H80D	109.5
C38—C37—H37A	109.0	C79A—C80A—H80E	109.5
C38—C37—H37B	109.0	C79A—C80A—H80F	109.5
C37—C38—H38A	108.5	H80D—C80A—H80E	109.5
C37—C38—H38B	108.5	H80D—C80A—H80F	109.5
C37—C38—C39	115.1 (10)	H80E—C80A—H80F	109.5
			
Ir1—N1—C1—C2	175.2 (7)	C26—C27—C28—C33	2.0 (10)
Ir1—N1—C5—C4	−173.2 (6)	C27—N3—C23—C24	−2.4 (9)
Ir1—N1—C5—C6	6.7 (7)	C27—C28—C29—Ir1	−3.7 (7)
Ir1—N2—C12—C13	177.5 (5)	C27—C28—C29—C30	178.9 (6)
Ir1—N2—C16—C15	−178.4 (4)	C27—C28—C33—C32	179.7 (6)
Ir1—N2—C16—C17	2.2 (6)	C28—C29—C30—C31	0.7 (10)
Ir1—N3—C23—C24	−178.9 (5)	C29—C28—C33—C32	−2.3 (10)
Ir1—N3—C27—C26	178.2 (5)	C29—C30—C31—C32	−0.7 (12)
Ir1—N3—C27—C28	−1.0 (6)	C29—C30—C31—C34	173.9 (7)
Ir1—C7—C8—C9	−175.6 (5)	C30—C31—C32—C33	−0.7 (12)
Ir1—C29—C30—C31	−176.2 (5)	C30—C31—C34—N4	141.8 (7)
Ir2—N5—C41—C42	−175.2 (5)	C31—C32—C33—C28	2.2 (11)
Ir2—N5—C45—C44	177.4 (5)	C32—C31—C34—N4	−43.7 (11)
Ir2—N5—C45—C46	−2.3 (6)	C33—C28—C29—Ir1	178.2 (5)
Ir2—N6—C52—C53	−172.1 (5)	C33—C28—C29—C30	0.8 (9)
Ir2—N6—C56—C55	172.4 (5)	C34—N4—C35—C36	−176.5 (6)
Ir2—N6—C56—C57	−6.4 (7)	C34—C31—C32—C33	−175.3 (7)
Ir2—N7—C63—C64	−176.5 (6)	C35—N4—C34—C31	−59.9 (9)
Ir2—N7—C67—C66	177.4 (5)	C35—C36—C37—C38	177.3 (9)
Ir2—N7—C67—C68	−3.0 (6)	C36—C37—C38—C39	−171.4 (10)
Ir2—C47—C48—C49	−175.7 (5)	C37—C38—C39—C40	−62.2 (18)
Ir2—C58—C59—C60	179.8 (5)	C41—N5—C45—C44	2.1 (8)
Ir2—C69—C70—C71	173.1 (5)	C41—N5—C45—C46	−177.7 (5)
N1—C1—C2—C3	0.3 (15)	C41—C42—C43—C44	−2.3 (11)
N1—C5—C6—C7	−1.3 (9)	C42—C43—C44—C45	4.1 (11)
N1—C5—C6—C11	177.6 (7)	C43—C44—C45—N5	−4.0 (10)
N2—C12—C13—C14	1.2 (11)	C43—C44—C45—C46	175.8 (6)
N2—C16—C17—C18	−179.1 (5)	C44—C45—C46—C47	−177.2 (6)
N2—C16—C17—C22	4.2 (7)	C44—C45—C46—C51	4.2 (10)
N3—C23—C24—C25	2.1 (10)	C45—N5—C41—C42	−0.4 (9)
N3—C27—C28—C29	3.1 (8)	C45—C46—C47—Ir2	−1.5 (7)
N3—C27—C28—C33	−178.8 (6)	C45—C46—C47—C48	−177.9 (5)
N4—C35—C36—C37	−176.9 (7)	C45—C46—C51—C50	177.4 (6)
N5—C41—C42—C43	0.5 (10)	C46—C47—C48—C49	0.2 (9)
N5—C45—C46—C47	2.5 (7)	C47—C46—C51—C50	−1.1 (10)
N5—C45—C46—C51	−176.0 (6)	C47—C48—C49—C50	−0.7 (10)
N6—C52—C53—C54	0.0 (12)	C48—C49—C50—C51	0.2 (12)
N6—C56—C57—C58	2.6 (8)	C49—C50—C51—C46	0.7 (12)
N6—C56—C57—C62	−174.3 (6)	C51—C46—C47—Ir2	177.1 (5)
N7—C63—C64—C65	−1.7 (12)	C51—C46—C47—C48	0.7 (9)
N7—C67—C68—C69	0.6 (8)	C52—N6—C56—C55	−3.2 (9)
N7—C67—C68—C73	178.3 (6)	C52—N6—C56—C57	178.1 (6)
N8—C75—C76—C77	−165.8 (10)	C52—C53—C54—C55	−2.5 (12)
N8—C75—C76—C77A	161.9 (11)	C53—C54—C55—C56	2.1 (12)
C1—N1—C5—C4	4.6 (11)	C54—C55—C56—N6	0.7 (10)
C1—N1—C5—C6	−175.5 (6)	C54—C55—C56—C57	179.3 (7)
C1—C2—C3—C4	−0.5 (16)	C55—C56—C57—C58	−176.0 (6)
C2—C3—C4—C5	2.8 (16)	C55—C56—C57—C62	7.1 (10)
C3—C4—C5—N1	−4.9 (13)	C56—N6—C52—C53	2.8 (10)
C3—C4—C5—C6	175.2 (9)	C56—C57—C58—Ir2	2.7 (7)
C4—C5—C6—C7	178.6 (7)	C56—C57—C58—C59	−178.3 (6)
C4—C5—C6—C11	−2.4 (12)	C56—C57—C62—C61	177.7 (7)
C5—N1—C1—C2	−2.4 (12)	C57—C58—C59—C60	1.0 (10)
C5—C6—C7—Ir1	−5.0 (8)	C58—C57—C62—C61	1.0 (11)
C5—C6—C7—C8	176.5 (6)	C58—C59—C60—C61	−0.3 (12)
C5—C6—C11—C10	−178.5 (7)	C59—C60—C61—C62	−0.2 (13)
C6—C7—C8—C9	2.6 (9)	C60—C61—C62—C57	−0.1 (13)
C7—C6—C11—C10	0.4 (11)	C62—C57—C58—Ir2	179.7 (5)
C7—C8—C9—C10	−0.7 (10)	C62—C57—C58—C59	−1.4 (9)
C8—C9—C10—C11	−1.4 (11)	C63—N7—C67—C66	−0.6 (9)
C9—C10—C11—C6	1.6 (12)	C63—N7—C67—C68	179.0 (6)
C11—C6—C7—Ir1	176.0 (6)	C63—C64—C65—C66	1.4 (12)
C11—C6—C7—C8	−2.4 (10)	C64—C65—C66—C67	−0.8 (11)
C12—N2—C16—C15	−0.2 (8)	C65—C66—C67—N7	0.4 (10)
C12—N2—C16—C17	−179.6 (5)	C65—C66—C67—C68	−179.2 (6)
C12—C13—C14—C15	−1.5 (11)	C66—C67—C68—C69	−179.8 (6)
C13—C14—C15—C16	0.9 (11)	C66—C67—C68—C73	−2.1 (10)
C14—C15—C16—N2	−0.1 (10)	C67—N7—C63—C64	1.3 (10)
C14—C15—C16—C17	179.2 (6)	C67—C68—C69—Ir2	2.2 (7)
C15—C16—C17—C18	1.6 (9)	C67—C68—C69—C70	179.2 (5)
C15—C16—C17—C22	−175.1 (6)	C67—C68—C73—C72	−176.8 (6)
C16—N2—C12—C13	−0.4 (10)	C68—C69—C70—C71	−3.6 (9)
C16—C17—C18—C19	−173.0 (6)	C69—C68—C73—C72	0.7 (10)
C16—C17—C22—Ir1	−9.0 (6)	C69—C70—C71—C72	3.4 (10)
C16—C17—C22—C21	170.6 (5)	C69—C70—C71—C74	−175.5 (6)
C17—C18—C19—C20	1.4 (9)	C70—C71—C72—C73	−1.0 (10)
C18—C17—C22—Ir1	174.3 (4)	C70—C71—C74—N8	88.1 (8)
C18—C17—C22—C21	−6.2 (8)	C71—C72—C73—C68	−1.0 (11)
C18—C19—C20—C21	−3.4 (9)	C72—C71—C74—N8	−90.9 (8)
C19—C20—C21—C22	0.5 (9)	C73—C68—C69—Ir2	−175.6 (5)
C20—C21—C22—Ir1	−176.4 (4)	C73—C68—C69—C70	1.5 (9)
C20—C21—C22—C17	4.2 (8)	C74—N8—C75—C76	−176.3 (7)
C22—C17—C18—C19	3.6 (9)	C74—C71—C72—C73	178.0 (7)
C23—N3—C27—C26	1.3 (9)	C75—N8—C74—C71	−58.0 (8)
C23—N3—C27—C28	−177.9 (5)	C75—C76—C77—C78	−178.8 (13)
C23—C24—C25—C26	−0.6 (10)	C75—C76—C77A—C78A	−165.5 (15)
C24—C25—C26—C27	−0.3 (10)	C76—C77—C78—C79	167.4 (15)
C25—C26—C27—N3	0.0 (10)	C76—C77A—C78A—C79A	−78 (2)
C25—C26—C27—C28	179.1 (6)	C77—C78—C79—C80	55 (3)
C26—C27—C28—C29	−176.1 (6)	C77A—C78A—C79A—C80A	−71 (3)

### Hydrogen-bond geometry (Å, º)

Cg1–Cg6 are the centroids of the C6–C11, N2/C12–C16, C17–C22, C46–C51, C57–C62 and C68–C73 rings, respectively.

**Table:**

*D*—H···*A*	*D*—H	H···*A*	*D*···*A*	*D*—H···*A*
N4—H4*A*···Cl2^i^	0.89	2.30	3.172 (6)	168
N4—H4*B*···Cl1^i^	0.89	2.26	3.142 (6)	172
N8—H8*A*···Cl2	0.89	2.21	3.073 (6)	165
N8—H8*B*···Cl1	0.89	2.16	3.044 (6)	171
C20—H20···*Cg*1^ii^	0.93	3.12	3.497 (7)	145
C24—H24···*Cg*4^iii^	0.93	2.89	3.532 (7)	139
C26—H26···*Cg*5	0.93	2.79	3.645 (7)	158
C34—H34*B*···*Cg*3^ii^	0.97	2.91	3.422 (7)	160
C37—H37···*Cg*1^ii^	0.97	3.01	3.818 (7)	141
C49—H49···*Cg*2^iii^	0.93	3.07	3.705 (7)	145
C53—H53···*Cg*6^iii^	0.93	3.10	3.692 (7)	135
C65—H65···*Cg*3	0.93	2.86	3.530 (7)	135

Symmetry codes: (i) *x*+1, −*y*+1/2, *z*+1/2; (ii) −*x*+2, −*y*+1, −*z*+1; (iii) *x*, −*y*+1/2, *z*−3/2.
